# Student Learning Behavior Recognition Incorporating Data Augmentation with Learning Feature Representation in Smart Classrooms

**DOI:** 10.3390/s23198190

**Published:** 2023-09-30

**Authors:** Zhifeng Wang, Longlong Li, Chunyan Zeng, Jialong Yao

**Affiliations:** 1 Faculty of Artificial Intelligence in Education, Central China Normal University, Wuhan 430079, China; 2 Hubei Key Laboratory for High-Efficiency Utilization of Solar Energy and Operation Control of Energy Storage System, Hubei University of Technology, Wuhan 430068, China

**Keywords:** teaching evaluation system, student learning behavior, data augmentation, smart classrooms

## Abstract

A robust and scientifically grounded teaching evaluation system holds significant importance in modern education, serving as a crucial metric that reflects the quality of classroom instruction. However, current methodologies within smart classroom environments have distinct limitations. These include accommodating a substantial student population, grappling with object detection challenges due to obstructions, and encountering accuracy issues in recognition stemming from varying observation angles. To address these limitations, this paper proposes an innovative data augmentation approach designed to detect distinct student behaviors by leveraging focused behavioral attributes. The primary objective is to alleviate the pedagogical workload. The process begins with assembling a concise dataset tailored for discerning student learning behaviors, followed by the application of data augmentation techniques to significantly expand its size. Additionally, the architectural prowess of the Extended-efficient Layer Aggregation Networks (E-ELAN) is harnessed to effectively extract a diverse array of learning behavior features. Of particular note is the integration of the Channel-wise Attention Module (CBAM) focal mechanism into the feature detection network. This integration plays a pivotal role, enhancing the network’s ability to detect key cues relevant to student learning behaviors and thereby heightening feature identification precision. The culmination of this methodological journey involves the classification of the extracted features through a dual-pronged conduit: the Feature Pyramid Network (FPN) and the Path Aggregation Network (PAN). Empirical evidence vividly demonstrates the potency of the proposed methodology, yielding a mean average precision (mAP) of 96.7%. This achievement surpasses comparable methodologies by a substantial margin of at least 11.9%, conclusively highlighting the method’s superior recognition capabilities. This research has an important impact on the field of teaching evaluation system, which helps to reduce the burden of educators on the one hand, and makes teaching evaluation more objective and accurate on the other hand.

## 1. Introduction

The rapid advancements in computer technologies, such as artificial intelligence, big data, and cloud computing, have led to the pervasive integration of smart classrooms in learning and teaching [1,2,3]. To bolster the development of these smart classrooms, the establishment of a robust and multifaceted teaching evaluation system is imperative [4,5], with a primary focus on comprehensive teaching evaluations. Among the array of evaluation tools, the recognition of student learning behaviors emerges as a particularly potent method for assessing teaching approaches [6,7]. By scrutinizing and identifying student learning behaviors, educators gain valuable insights into their students’ progress and learning efficacy, enabling them to fine-tune teaching strategies and methods accordingly. This ultimately cultivates a more conducive learning environment [8].

In traditional classroom settings, educators and teaching personnel primarily rely on direct observation of students’ learning behaviors to comprehend their ongoing learning trajectory [9,10,11]. Additionally, indirect evaluation methods, including grade records, homework completion, and class attendance tracking, are harnessed to gauge students’ learning journey. With the integration of multimedia teaching in classrooms, sensor-based technologies have emerged, enabling the nuanced recognition of students’ learning behaviors. For instance, Mohammed et al. [12] harnessed the Internet of Things (IoT) to devise an automated classroom behavior classification system. This system assesses class effectiveness through result recognition, furnishing administrators with insightful data to enhance overall classroom performance. In the field of psychology, researchers have explored the benefits of MOOC learning for students with dependent speech cognitive style and dependent image cognitive style based on students’ brain waves, so as to provide targeted guidance for students’ learning styles. However, traditional direct observation methods rely too much on the subjectivity of the observer and are time-consuming, while sensor technology and brainwave observation rely on expensive equipment and are difficult to popularize [13].

With the maturity of computer vision technology, it has become a trend to apply it to the intelligent classroom environment as an auxiliary tool for teaching evaluation. These technologies are characterized by continuous monitoring, objective evaluation and real-time interaction. Wang [14] proposed a deep residual network that leverages residual structures to identify students’ engagement levels during class. This technological innovation serves as a pivotal cornerstone for the development of intelligent classrooms. It is worth noting that researchers in the field of education and teaching have recognized that factors such as different lighting conditions, different viewing angles, and the quality of image acquisition equipment in dense teaching environments pose challenges for the accurate identification of student learning behaviors [15,16,17]. Therefore, in addition to improving lighting facilities and optimizing the layout of camera equipment, the researchers are also working to develop and optimize more universal detection algorithms to address these challenges.

Our research question can be summarized as follows: how to accurately detect and recognize students’ learning behaviors in complex smart classrooms (including a large number of students, occlusions, and different observation angles)? Building upon the foundation of previous research, this paper adopts the YOLOv7 framework as the principal network architecture, thereby achieving swift recognition while preserving accuracy. Moreover, we seamlessly integrate the attention recognition mechanism of CBAM to heighten recognition precision within complex background scenarios. Concretely, we employ the proposed algorithm to detect seven distinctive learning behaviors typically exhibited by elementary school students, encompassing actions like writing, reading, raising hands, and participating in discussions. We undertake a comprehensive performance comparison against state-of-the-art (SOTA) learning behavior recognition frameworks. This paper makes several significant contributions that advance the field of student learning behavior recognition:

- In response to the absence of specialized datasets for student learning behavior recognition in the field of education, this study introduces the Student Learning Behavior (SLB) dataset. This dataset is meticulously annotated to include comprehensive information about classroom learning behaviors among primary and secondary school students. Researchers and practitioners can access this valuable resource on GitHub through the following link: https://github.com/houhou34/Teaching-evaluation-dataset (accessed on 6 March 2023). The availability of this dataset addresses a critical gap in the domain, facilitating the development and evaluation of learning behavior recognition models.
- This paper proposes a novel learning behavior recognition method that builds upon the YOLOv7 architecture. This method has been designed to accurately detect multiple students’ learning behaviors. Furthermore, we conduct an in-depth investigation into the impact of incorporating the attention detection mechanism into our proposed method. This integration of the attention mechanism is shown to significantly enhance the recognition performance of our model.
- Our developed student learning behavior recognition network demonstrates a notable improvement over state-of-the-art (SOTA) methods. Specifically, it achieves an enhanced mean average precision (mAP) by 11.9%. Our model exhibits remarkable advantages in detecting students, even in scenarios with significant occlusion. It excels in identifying common classroom behaviors such as students turning their heads, raising their hands, and looking up. These achievements underscore the effectiveness of our proposed approach in real-world classroom environments.
- We tackle the challenge posed by limited training samples by employing two crucial techniques: data augmentation and transfer learning. These methods allow us to maximize the utility of the available data, enhancing the robustness and generalization capacity of our model.

The ensuing sections of this paper are structured as follows: Section 2 offers an in-depth review of pertinent research in student classroom behavior recognition and the YOLO algorithm. In Section 3, we provide a problem definition of this research and summarize the notations that appear in this paper. Section 4 elucidates the procedural approach and network framework adopted for detecting and identifying student behaviors. In Section 5, we present a detailed comparison and conduct ablation experiments to rigorously assess the efficacy of our proposed methodology. Lastly, Section 6 encapsulates our conclusions and outlines potential avenues for future research exploration.

## 2. Related Work

In this section, we will take a closer look at student learning behavior and current methods for detecting student behavior in the classroom, as well as provide an overview of the YOLO object recognition model. In addition, the application of attention mechanisms in computer vision is explored.

### 2.1. Student Learning Behavior

The manifestations, characteristics, and phenomena apparent in students’ learning behaviors have been extensively studied in the fields of educational research and educational psychology [18]. Typically, these characteristics and phenomena are interrelated with various aspects of students’ cognitive, affective, and social interactions. The impact of students’ learning behaviors on their academic performance and learning experiences is significant. Learning analytics is a rapidly developing academic field that evaluates the status of students from various perspectives. In classroom instruction, attendance is vital, as it serves as the initial step towards participation in classroom activities and academic seminars [19]. Furthermore, attention plays a crucial role in gauging a student’s dedication to learning. Students must maintain a high level of focus to comprehend and absorb course content effectively. Effective attention and focus contribute significantly to academic performance. Collaboration with peers further underscores the pivotal role of cooperative learning proficiency when working in teams [20,21]. Cultivating positive study behaviors and habits facilitates meeting academic challenges, enhancing academic performance, and improving lifelong learning outcomes. Learning behaviors and habits are instrumental in the success of learners. Therefore, educators must foster these behaviors during the teaching and learning process to assist students in achieving better academic outcomes and preparing for future careers.

### 2.2. Student Learning Behavior Recognition Based on Deep Learning

Student behavior within the classroom significantly impacts the quality of education [7]. Active participation, attentive listening, note-taking, and adherence to classroom rules all contribute to effective teaching and a conducive learning environment [22,23]. To address the significance of student behaviors, researchers have harnessed deep learning techniques to construct frameworks for detecting and recognizing these behaviors. An enhanced Faster R-CNN model for student behavior recognition was proposed by Zheng et al. [24], which incorporated a novel scale-aware recognition head and a fresh feature fusion strategy for detecting low-resolution behaviors. Lv et al. [25] integrated the ResNet and FPN modules into the SSD model, addressing the challenge of recognizing small targets at the back of the classroom and significantly boosting image recognition efficiency. Mindoro et al. [26] introduced a method to predict student behavior based on facial expressions and real-time behavior recognition, implementing it with the YOLOv3 network. Tang et al. [27] employed a weighted bidirectional feature pyramid network (BIFPN) along with YOLOv5’s feature pyramid structure, effectively transforming the target recognition issue into a fine-grained representation challenge. Furthermore, they enhanced the non-maximal value suppression algorithm to improve the differentiation of highly occluded objects. Yang et al. [28] developed an analytical system for assessing students’ learning status, harnessing the YOLOv5 network and the CBAM attention module to extract robust features from student behavior. Mo et al. [29] proposed a multitask learning method to identify students’ classroom behaviors, which used MTHN module to extract intermediate heat maps and combined key points and object locations to simulate students’ behaviors. It is noteworthy that researchers in education and teaching face the challenge of detecting behaviors within densely populated classrooms, calling for a comprehensive solution to tackle missed and erroneous recognitions due to occlusion and observation angles during classroom behavior recognition.

In addition to computer vision-based techniques for identifying student classroom behaviors, Zhao et al. [30] leveraged deep learning and developed models grounded in Deep Belief Networks (DBNs) to assess teaching speech standards. Lu et al. [31] applied feature data mining methods to detect learning behaviors in online English classrooms. In Table 1, comparisons of deep learning-based student learning behavior recognition methods in terms of methods, implementation techniques, and results are shown.

### 2.3. Target Detection Based on YOLO

This subsection provides an extensive overview of the YOLO networks’ evolution, highlighting their multifarious applications in student learning behavior detection.

The You Only Look Once (YOLO) algorithm, introduced by Redmon et al. [32], represents a single-stage target detection approach. It reimagines target detection as a regression task, negating the need for candidate region extraction inherent in traditional two-stage target detection algorithms. The YOLO algorithm simultaneously ascertains target categories and regresses their positions using a singular network. Progressing iterations have yielded enhanced YOLO algorithm performance. YOLOv2 [33] introduced anchor boxes and batch normalization techniques to amplify detection accuracy. YOLOv3 [34] integrated the Darknet-53 architecture and Feature Pyramid Network (FPN) to elevate target detection efficacy. YOLOv4 [35] streamlined the target detection model by refining the training threshold. YOLOv5 [36] unveiled five distinct models of varying sizes, attaining performance amelioration through channel scaling and model size adjustments. Subsequently, YOLOv6 and YOLOv7 emerged. YOLOv6 embraced the RepVGG architecture, augmenting GPU device adaptability and engineering adaptations [37]. YOLOv7 incorporated module re-referencing and dynamic tag assignment strategies, bolstering both speed and accuracy, effectively outpacing existing target detectors in the 5 FPS to 160 FPS range [38].

Researchers have harnessed the YOLO family of algorithms across diverse applications within the realm of student behavior detection. Chen et al. [39] introduced an enhanced YOLOv4 behavior detection algorithm infused with Repulsion loss functions, thus amplifying detection capabilities for varying behaviors in the classroom. Mindoro et al. [26] utilized the YOLOv3 algorithm to decipher students’ facial expressions and predict their behaviors. Wei and Ding [40] harnessed the OpenPose algorithm to extract global features of the human body and joint angle information, effectively distinguishing head-up from head to down states. Additionally, they employed the YOLO algorithm to discern hand-related information, determining whether a student was using a cellphone.

### 2.4. Attention Mechanism in Computer Vision

The attention mechanism, often referred to as selective attention, constitutes a cognitive ability observed in both humans and animals [41]. This ability enables the selective focusing on specific information while effectively ignoring irrelevant data when encountering intricate stimuli [42]. Such a mechanism serves to enhance cognitive efficiency and accuracy, thereby facilitating more streamlined information processing. The study of attention mechanisms finds application across a diverse array of fields, spanning cognitive psychology, neuroscience, and computer science. In the domain of clinical neuroscience, attention mechanisms are explored for their potential in diagnosing and addressing attention-related disorders, such as ADHD [43]. Within the realm of speech recognition, attention mechanisms find employment in critical tasks such as speech recognition and synthesis. Moreover, attention mechanisms have garnered substantial acceptance and utilization in recommendation systems, computer vision, and speech recognition, leading to tangible enhancements in model performance. In recommendation systems, these mechanisms prove invaluable in deciphering user interests and preferences, subsequently refining the accuracy of recommendations. Within the scope of computer vision, attention mechanisms are strategically deployed for tasks encompassing image classification, target recognition, and image generation, culminating in augmented model performance by virtue of their capacity to concentrate on pertinent image regions [44].

Specifically within the realm of computer vision, attention mechanisms can be broadly categorized into three distinctive types: channel attention mechanisms, spatial attention mechanisms, and region attention mechanisms. Channel attention mechanisms allocate distinct weights to individual channels within the feature map, thus amplifying recognition precision and efficiency. A salient illustration of channel attention mechanisms is the Squeeze-and-Excitation module as seen in SENet, which autonomously discerns the significance of each channel in the feature map, invariably heightening recognition accuracy [45]. Spatial attention mechanisms, on the other hand, endow diverse spatial locations within the feature map with varying weights, optimizing recognition precision and efficiency. The Spatial Attention module, a prime example of a spatial attention mechanism within CBAM, effectively captures the significance of each spatial location in the feature map, resulting in an elevated level of recognition accuracy [46]. Region attention mechanisms further contribute by assigning distinct weights to discrete regions within an image, thereby enhancing recognition accuracy and efficiency. Notably, RoI Pooling as implemented in Faster R-CNN exemplifies a region attention mechanism, effectuating the extraction of feature maps of fixed dimensions through pooling operations applied to regions of interest [47]. These various attention mechanisms collectively bolster performance and precision across a spectrum of computer vision tasks.

Overall, the integration of attention mechanisms across diverse domains, including the realm of computer vision, holds substantial potential for elevating model performance and refining the concentration on pertinent information. By harnessing the capabilities of attention mechanisms, researchers have made substantial strides in areas such as student behavior recognition, target recognition, and related tasks. These developments pave the way for further exploration and refinement of attention-based models within the dynamic landscape of computer vision.

**Summary**: YOLO networks have attained widespread utilization and notable advancements in the arena of student learning behavior recognition. They have demonstrated remarkable efficacy in capturing localized visual features, such as body pose and facial expressions, that are integral for behavior recognition. Nonetheless, YOLO networks might encounter challenges in effectively modeling intricate relationships existing between diverse body parts and in comprehensively capturing overarching contextual information. Hence, attention mechanisms emerge as a prospective avenue to enhance the capabilities of student learning behavior recognition systems. These mechanisms possess the inherent capacity to apprehend global dependencies and contextual nuances. By adeptly modeling relationships between distinct body parts and judiciously considering the holistic context of students’ classroom behaviors, we stand poised to amplify both the accuracy and the resilience of behavior recognition models.

## 3. Preliminaries

This section aims to elucidate the task of identifying student learning behaviors and the behavior recognition framework adopted in this study. To facilitate understanding, Table 2 presents crucial symbols alongside their corresponding interpretations.

### 3.1. Problem Definition

**Figure 1:**
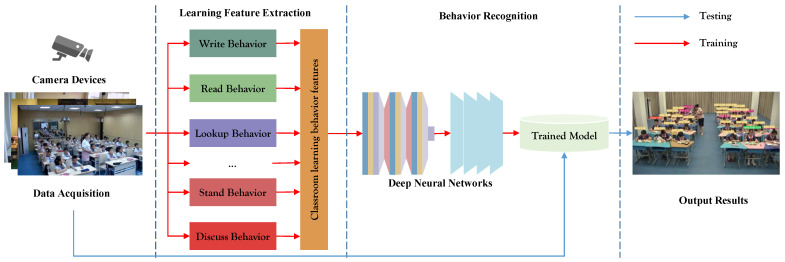
Process of student learning behavior recognition.

The central challenge tackled pertains to the detection and recognition of student learning behaviors within classroom settings. Figure 1 provides an overview of the student classroom learning behavior testing system’s workflow adopted in this research. The red and blue arrows symbolize the training and testing processes, correspondingly. The system starts with continuous video frame input from a classroom camera. It unfolds through three principal stages: the collection of student classroom learning behavior data, feature extraction, and behavior detection and recognition.

### 3.2. Definitions

**Definition** **1.** *(Data collection of student learning behavior): Continuous video frames are captured by classroom cameras.*

**Definition** **2.** *(Feature extraction of student learning behavior): The feature vectors corresponding to diverse student learning behaviors are extracted via deep neural network. These feature vectors are subsequently employed as inputs for the feature detection network during both the training and testing phases of the proposed network.*

**Definition** **3.** *(Detection of student learning behavior characteristics): The proposed network amalgamates the CBAM attention mechanism with the YOLOv7 feature detection network to construct a model geared towards detecting and recognizing student classroom learning behaviors.*

## 4. Methods

This section provides details of the framework for analyzing student learning behavior. The task of detecting and recognizing student behavior within an smart classroom teaching environment is complex due to challenges such as diverse illumination sources, lighting variations, background interference, occlusion, and image noise. The You Only Look Once (YOLO) algorithm, known for its real-time performance, global sensing capability, multi-target recognition, and end-to-end training, has gained prominence in target recognition. Leveraging its versatility, this study presents a learning behavior recognition framework centered around YOLOv7.

### 4.1. Network Design of Student Behavior Recognition Task

**Figure 2:**
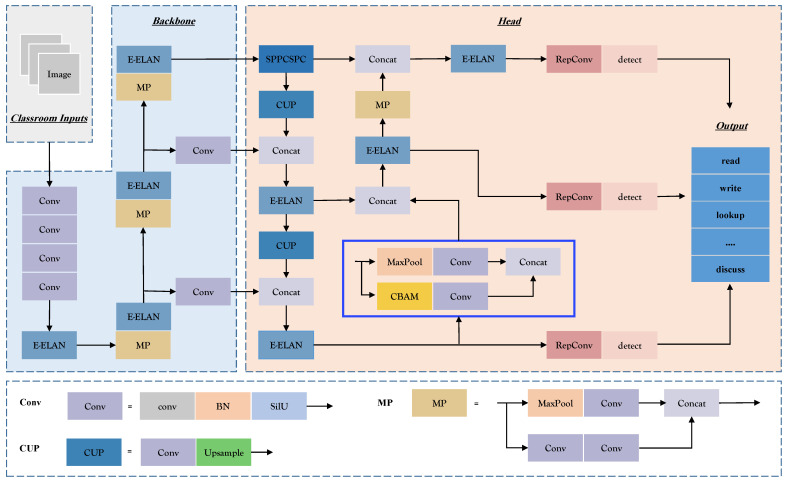
Student learning behavior recognition framework.

The network structure adopted for this research is divided into four main components: Input, Backbone, Head, and Output. The input image is resized to 640 × 640 pixels and enters the backbone network. The head network generates three tiers of feature maps with varying dimensions. The RepConv module’s recognition output yields both the target object’s position and category information. The location information is typically represented as bounding box coordinates [33], where ($t_{x}$, $t_{y}$) denote the center coordinates, and ($t_{w}$, $t_{h}$) represent the bounding box’s width and height. The category information is deduced using a multi-category classifier, obtaining confidence levels. This indicates the presence of the target object within the bounding box and provides a measure of accuracy. The method employs three anchor frames, resulting in 36 outputs per layer, computed as (7+5)×3. These outputs are then concatenated with the feature map size to produce the final output. Figure 2 visually outlines the network structure, with blue boxes signifying enhancements made.

The backbone network, as depicted in Figure 2, incorporates the Conv module for input image normalization and nonlinear transformation. The MaxPool (MP) module and the E-ELAN module work together to learn image features with varying perceptual fields. The outcomes from three distinct E-ELANs are combined into the YOLOv7 feature detection network. The integration of the E-ELAN module enhances YOLOv7’s learning capabilities, parameter utilization, and computational efficiency. To cater to targets of different scales, the head network augments feature map output from the backbone network using the Spatial Pyramid Pooling Combined with Spatial Context Prediction (SPPCSPC) module [38]. Furthermore, the E-ELAN module is employed to further heighten the network’s computational efficiency.

This paper introduces the Convolutional Block Attention Module (CBAM) attention mechanism to underscore critical information concerning students’ learning behaviors. CBAM effectively captures contextual features and bolsters the network’s feature detection capabilities. Figure 2 illustrates the network architecture integrating the CBAM attention mechanism. The network’s prediction results, post the RepConv module, comprise the multi-scale output from the head network.

### 4.2. Extended-Efficient Layer Aggregation Networks Module

**Figure 3:**
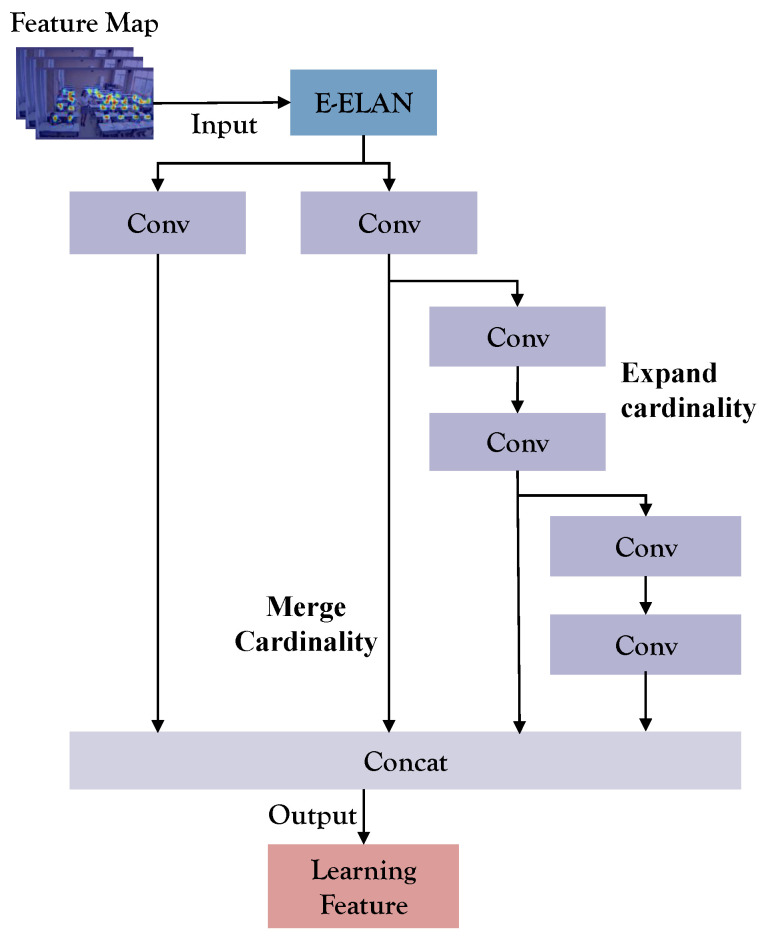
E-ELAN module’s architectural depiction.

This model introduces the Extended-efficient layer aggregation networks (E-ELAN) module, elevating network learning potential through the Expand, Shuffle, Merge Cardinality Network (EALN) approach. The E-ELAN module modifies both the backbone network and the head network’s structure [38]. Group convolution is employed to expand the feature base count, and features from different groups are fused using shuffling and merging cardinality operations. This strategy improves parameter utilization, computational efficiency, and features learned from various feature maps. Figure 3 illustrates the architecture of the E-ELAN module.

### 4.3. Convolutional Block Attention Module

**Figure 4:**
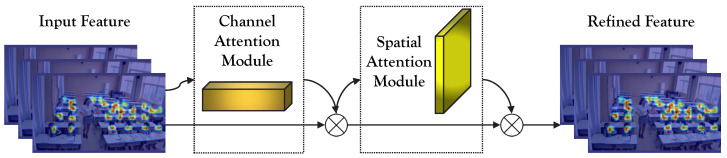
Schematic representation of the CBAM module.

The Convolutional Block Attention Module (CBAM) is an attention mechanism commonly employed in convolutional neural networks to enhance model performance in tasks such as recognition and classification, as shown in Algorithm 1. Its primary function is to adapt the input feature map by selectively highlighting crucial features through attention tuning across both channel and spatial dimensions. By doing so, the CBAM mechanism effectively improves the network’s ability to understand and model complex images. The structure of the CBAM module is visually represented in Figure 4.

The CBAM module is composed of two distinct sub-modules: the Channel Attention Module (CAM) and the Spatial Attention Module (SAM). The CAM’s role is to perform spatially informative aggregation on the feature map, utilizing global max pooling and global average pooling techniques. Subsequently, the resulting feature maps undergo a two-layer Multi-Layer Perceptron (MLP) neural network. The output features are then element-wise summed and passed through a sigmoid activation function, ultimately producing the final channel attention feature.
**Algorithm 1:** Algorithm for CBAM attention mechanism**Input:** Network intermediate volume characteristics map**Output:** Attention maps  1: CAM performs a spatially informative aggregation operation on the feature map:
$$\begin{array}{cc} M_{c}\left(F\right) & =\sigma (\mathrm{MLP}(\mathrm{AvgPool}(F))+(\mathrm{MLP}(\mathrm{MaxPool}(F)))\;\\ & =\sigma \left(W_{1}\left(W_{0}\left(F_{avg}^{c}\right)\right)+W_{1}\left(W_{0}\left(F_{\max}^{c}\right)\right)\right) \end{array}$$  2: The channel attention map is multiplied at the pixel level with the original image:
$$\begin{array}{cc} F^{\prime }= & M_{c}\left(F\right)⊗F, \end{array}$$  3: SAM performs feature focus and dimensionality reduction operations on feature maps:
$$\begin{array}{cc} M_{s}\left(F\right) & =\sigma \left(f^{7\times 7}\left(\left[\mathrm{AvgPool}\left(F^{\prime }\right);\mathrm{Max}\mathrm{Pool}\left(F^{\prime }\right)\right]\right)\right)\;\\ & =\sigma \left(f^{7\times 7}\left(\left[F_{avg}^{s};F_{\max}^{s}\right]\right)\right) \end{array}$$  4: Spatial Attention Maps are multiplied by the original map at the pixel level:
$$\begin{array}{c} F^{\prime \prime }=M_{s}\left(F^{\prime }\right)⊗F^{\prime } \end{array}$$  5: Output CBAM processed feature maps.

SAM, on the other hand, operates on the feature map obtained from the CAM. It undertakes global max pooling and global average pooling along the channel dimension to yield two distinct feature maps. These feature maps are used to calculate the spatial attention feature using a sigmoid activation function. Finally, the spatial attention feature is element-wise multiplied with the initial feature map to generate the ultimate feature output.

The combined utilization of these modules, namely E-ELAN and CBAM, significantly contributes to the enhancement of object recognition models by bolstering the network’s learning capacity and selectively highlighting important features. CBAM attention mechanism can enhance CNN’s attention to important student behavior features, focus attention on features related to student behavior, and resist redundant information in the data. In addition, the CBAM attention mechanism can reduce unnecessary computational burden, filter out irrelevant feature information.

### 4.4. Data Augmentation

The efficacy of deep learning in the realm of computer vision hinges on access to extensive, meticulously annotated datasets [48]. However, constructing a high-quality dataset for target recognition introduces a host of challenges:

**(1) Labeling complexity**: Target recognition mandates the precise identification and classification of each object, rendering the annotation process more time-intensive. Ensuring accuracy and consistency necessitates skilled annotators. Moreover, complexities like occlusion, rotation, and pose variations further compound the labeling process.

**(2) Dataset imbalance**: Target recognition datasets often exhibit significant disparities in the number of samples across different categories, leading to suboptimal recognition for certain categories. It is crucial to achieve a balanced distribution of samples to alleviate category imbalance during dataset creation.

**(3) Dataset diversity**: The target recognition dataset must encompass a wide spectrum of characteristics—varied target objects, scenes, lighting conditions, poses, and viewpoints—to bolster generalization capabilities. Yet, curating such diverse datasets entails substantial investments of time and labor.

**(4) Dataset size**: Successful target recognition models typically demand a substantial volume of training data for optimal performance. However, constructing a sufficiently large dataset poses challenges due to the heightened complexity and time required for annotation.

**(5) Dataset quality**: Producing a high-quality target recognition dataset mandates annotators with professional acumen to ensure precision and uniformity. Moreover, datasets may inadvertently contain errors or noise, necessitating thorough screening and cleaning to preserve data quality.

To address these challenges, this paper turns to data augmentation, an essential technique to bolster the training of deep learning models using limited effective training data. Data augmentation diversifies the dataset, mitigates overfitting, and enhances the model’s capacity for generalization. In this study, data augmentation is performed on the self-built Student Learning Behavior (SLB) dataset through a series of techniques.

#### 4.4.1. Random Rotation Enhancement

**Figure 5:**
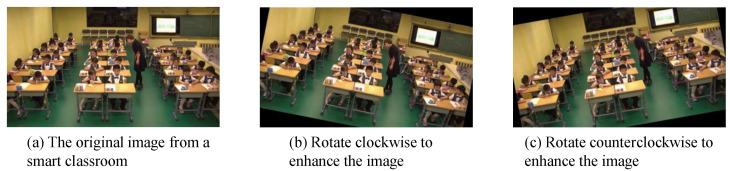
Illustration of random rotation enhancement.

To enhance the variability of the SLB dataset and mitigate the risk of overfitting, random rotation enhancement is introduced. This technique introduces diversity by applying random rotations to images. Figure 5 visually illustrates this process, depicting the original image, a 15° clockwise rotation, and a 15° counterclockwise rotation. Algorithm 2 provides the underlying principle behind random rotation enhancement.
**Algorithm 2:** Image random rotation enhancement algorithm**Input:** image max_angle**Output:** enhanced_image  1:  **function** random_rotation(image, max_angle)  2:  height, width = image.shape  3:  Randomly generate the rotation angle. Randomly generated rotation angle θ can be represented by the random number function rand:
$$\begin{array}{c} \theta =\mathrm{rand}\times 2\times \underline{\max}\mathrm{angle}-\underline{\max}\mathrm{angle} \end{array}$$  4:  Calculate the center of rotation. Assume that the coordinates of the center of rotation are (x_center,y_center).
$$\begin{array}{c} center\_x=\frac{width}{2},\;center\_y=\frac{height}{2} \end{array}$$  5:  Define the rotation matrix.
$$\begin{array}{c} \mathrm{rotation\_matrix}=\left[\begin{array}{ccc} \cos(\theta ) & -\sin(\theta ) & (1-\cos(\theta ))\cdot \mathrm{center\_x}+\sin(\theta )\cdot \mathrm{cente}\\ \sin(\theta ) & \cos(\theta ) & -\sin(\theta )\cdot \mathrm{center\_x}+(1-\cos(\theta ))\cdot \mathrm{cent}\\ 0 & 0 & 1 \end{array}\right] \end{array}$$  6:  Performs image rotation. The position of each pixel after rotation is ($x^{\prime }$,$y^{\prime }$).
$$\begin{array}{c} (x^{\prime },y^{\prime })=\left\{\begin{array}{c} x^{\prime }=\left(x-\mathrm{center\_x}\right)\cdot \cos\left(\theta \right)-\left(y-\mathrm{center\_y}\right)\cdot \sin\left(\theta \right)+\mathrm{center\_x}\\ y^{\prime }=\left(x-\mathrm{center\_x}\right)\cdot \sin\left(\theta \right)+\left(y-\mathrm{center\_y}\right)\cdot \cos\left(\theta \right)+\mathrm{center\_y} \end{array}\right. \end{array}$$  7:  **return** rotated_image  8:  **end function**

#### 4.4.2. Grayscale Enhancement

In the context of smart classroom environments, certain image details and features often pose challenges for human observation and automated recognition, particularly when influenced by low light conditions or shadows. Enhancing the grayscale level of an image serves to accentuate these intricate aspects, thereby bolstering the differentiation of features within the image. Moreover, grayscale enhancement transcends the realm of object color and encompasses other crucial cues like shape and edges.

Within this paper, we employ grayscale enhancement to the Single-Label Behavior (SLB) dataset. The technique involves extending the grayscale dynamic range of the original image to a predefined interval, facilitated through a linear relationship equation. The equation reads as follows, where f(x,y) and g(x,y) denote the grayscale values of pixels at positions (x,y) before and after enhancement. The parameters *a* and *b* stand for the minimum and maximum values of grayscale levels in the original image, while *c* and *d* pertain to the minimum and maximum values in the enhanced image:

(1) $$g\left(x,y\right)=\left\{\begin{array}{cc} c, & \left[0,a\right)\\ \frac{d-c}{b-a}\times f\left(x,y\right)+c, & \left[a,b\right]\\ d, & \left(b,255\right] \end{array}\right.$$

**Figure 6:**
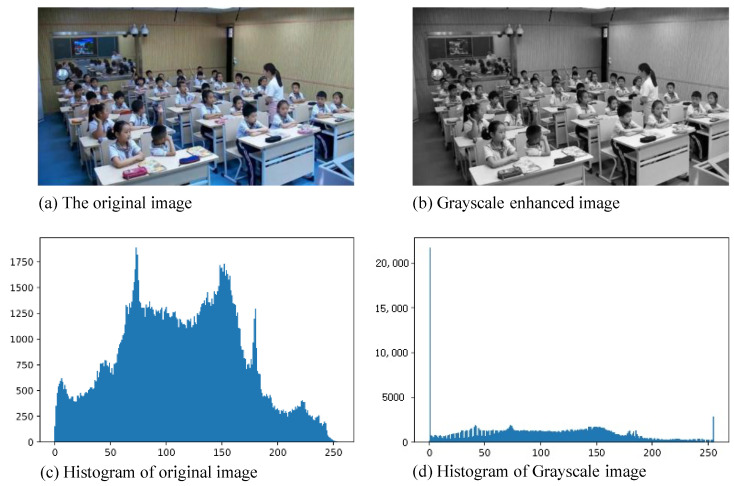
Illustration of grayscale enhancement.

This study embraces a grayscale transformation employing k=25% on the SLB dataset. An exemplar of the grayscale enhancement process is portrayed in Figure 6.

#### 4.4.3. Noise Enhancement

Within real classroom scenarios, images often encounter inevitable disturbances and noise, encompassing phenomena like lighting variations, image blurring, and image distortion. To bolster the model’s aptitude for discerning robust features and enhancing its resilience against disturbances and noise, this paper introduces noise to the images through a noise enhancement algorithm grounded in the mean filter approach.

**Figure 7:**
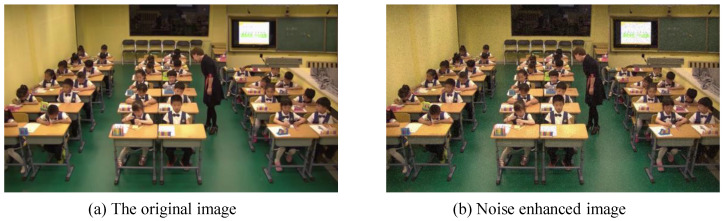
Noise enhancement example.

Figure 7 offers a visual demonstration of this transformation. In this context, the mean filter operates by replacing the value of the central pixel with the average of the pixel values within a window centered on the pixel. Typically, the size of the filter’s window corresponds to an odd number, determining its dimensions. The specifics of the image noise enhancement algorithm, utilizing mean filtering, are elucidated in Algorithm 3:
**Algorithm 3:** Image noise enhancement algorithm based on mean filtering**Input:** image kernel_size**Output:** enhanced_image  1:  **function**enhance_noise_with_average_filter(image, kernel_size)  2:  height, width = image.shape  3:  Creates a blank image of the same size as the original image.  4:  Gets the height and width of the image.  5:  Performs noise enhancement on the image.  6:  **for** y = 0 to height − 1 **do**  7:   **for** x = 0 to width − 1 **do**  8:      Calculate the boundary coordinates of the filter. Assume that the coordinates of the computational boundary are (x,y).
$$\begin{array}{c} \left(x.y\right)=\left\{\begin{array}{c} \mathrm{top}=\max(y-\lfloor N/2\rfloor ,0)\\ \mathrm{bottom}=\min(y+\lfloor N/2\rfloor ,\mathrm{height}-1)\\ \mathrm{left}=\max(x-\lfloor N/2\rfloor ,0)\\ \mathrm{right}=\min(x+\lfloor N/2\rfloor ,\mathrm{width}-1) \end{array}\right. \end{array}$$  9:      The average value is calculated for the pixels within the filter.
$$\begin{array}{c} {\mathrm{sum}}_{-}\mathrm{value}=\sum_{i=\mathrm{top}}^{\mathrm{bottom}}\sum_{j=\mathrm{left}}^{\mathrm{right}}\mathrm{image}(x+i-\lfloor N/2\rfloor ,y+j-\lfloor N/2\rfloor ) \end{array}$$ 10:      The average value is used as the enhanced pixel value.
$$\begin{array}{c} \mathrm{average}=\frac{\mathrm{sum}\_\mathrm{value}}{N\times N} \end{array}$$ 11:   **end for** 12:  **end for** 13:  **return** enhanced_image 14:  **end function**

In this study, the SLB dataset is enriched through the noise enhancement algorithm based on mean filtering, thereby simulating an array of disturbances and noise scenarios.

## 5. Experimental Results and Analysis

Building upon the aforementioned framework for learning behavior recognition, we conducted empirical research using real smart classroom data.

### 5.1. Dataset for Experiments

In the realm of computer vision, a plethora of datasets have emerged to cater to diverse visual tasks. Examples include the MNIST dataset for digit recognition, the KITTI dataset for autonomous driving research, and the ADE20K dataset for scene understanding. These datasets have furnished researchers and developers with extensive image samples and annotated information, thereby enabling comprehensive investigations and algorithmic advancements tailored to specific vision tasks. At the intersection of computer vision technology and pedagogy lies the significant challenge of detecting and recognizing diverse learning behaviors exhibited by students.

**Figure 8:**
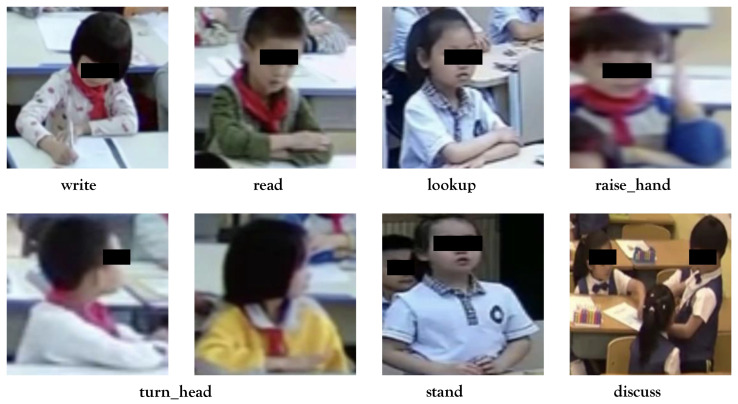
Example images from the SLB dataset.

In response to this challenge, we have crafted a dataset called Student Learning Behavior (SLB) to facilitate researchers and educators in comprehending students’ behavioral patterns and learning states. This dataset is constructed from classroom videos acquired from NPS, and comprises 600 high-resolution RGB color images, with one image extracted every 150 frames. Each image boasts dimensions of 2048 × 1152 pixels. The vott [49] open-source software was employed for annotating learning behaviors and bounding boxes of students in each image. The dataset encompasses seven categories of student classroom learning behaviors: write, read, lookup, turn_head, raise_hand, stand, and discuss. Furthermore, the dataset covers four smart classroom scenarios, each boasting distinct layouts. Within each scenario, approximately 30 individual students are present, culminating in around 120 distinct student objects. An illustrative instance of each behavior type is depicted in Figure 8. The dataset is divided into three segments: 420 images for training, 120 images for validation, and 60 images for testing. The distribution of different labels, conforming to the VOC2012 dataset format [38], is provided in Table 3.

### 5.2. Evaluation Metrics

To evaluate the effectiveness of the proposed approach, this study employs the PASCAL VOC metric [27]. This metric assesses the accuracy of object identification based on four key parameters: true positive (TP), true negative (TN), false positive (FP), and false negative (FN). TP signifies correct positive predictions, where both the predicted and true values are positive samples. TN denotes correct negative predictions, where both the predicted and true values are negative samples. FP represents incorrect positive predictions, where the predicted value is positive but the true value is negative. FN indicates incorrect negative predictions, where the predicted value is negative but the true value is positive. Precision and Recall are calculated based on these parameters.

(2) $$Precision=\frac{TP}{TP+FP}$$

(3) $$\begin{array}{c} Recall=\frac{TP}{TP+FN} \end{array}$$

Furthermore, the concepts of Average Precision (AP) and mean Average Precision (mAP) are introduced to jointly evaluate Precision and Recall. AP serves as a metric to assess the performance of detecting individual targets within specific categories. It quantifies the model’s accuracy by calculating the area under the precision-recall curve, as defined in Equation (4). AP values range from 0 to 1, with higher values indicating superior model performance. In contrast, mAP represents the mean Average Precision for detecting multiple targets across various categories. In multi-category target recognition tasks, an AP value can be computed for each category. These AP values are then averaged to derive mAP, as shown in Equation (5) [27].

In addition, this study uses Frames Per Second (FPS) to evaluate the real-time performance and efficiency of the model [27]. Its calculation method is given in Equation (6), where inference time refers to the time from the preprocessed image input model to the model output result, and NMS is the post-processing time.

(4) $$AP_{i}=\int_{0}^{1}P\left(r\right)dr$$

(5) $$mAP=\frac{1}{n}\sum_{i}^{n}\left(AP_{i}\right)$$

(6) FPS=1/in ferencetime+NMS

### 5.3. Baselines

In order to comprehensively evaluate the efficacy of our proposed method, we conducted a comparative analysis against several established models within the domain of student learning behavior recognition systems. Furthermore, we included YOLOv6, a recent addition to the YOLO family, as well as the lightweight YOLOv7-Tiny model from YOLOv7, for comparative purposes.

**SSD** [25]: This method employs the ResNet network for feature extraction and integrates the Region Proposal Network (RPN) for generating bounding boxes. Subsequently, a k-means algorithm is employed for post-processing and filtering.

**Faster R-CNN** [24]: Faster R-CNN, a classic two-stage target detection approach, is widely applied for various behavior detection tasks. It leverages Region Proposal Network (RPN) networks to streamline model computation and enhance detection efficiency.

**YOLOv3** [26]: YOLOv3-SPP employs a feature extraction network to capture features, followed by the utilization of the Spatial Pyramid Pooling (SPP) module for multi-scale feature extraction. The detection network is then utilized for behavior classification and positional regression to derive final behavior detection outcomes.

**YOLOv5** [27]: The YOLOv5 model adapts anchor frames during computation. It employs k-means clustering to determine n anchors and employs a genetic algorithm to randomize anchor width and height (wh). An anchor fitness approach is employed for evaluating obtained fitness.

**YOLOv6** [50]: YOLOv6 introduces varied backbone networks based on model scale and employs distinct activation functions for different scenarios to balance between field deployment and model accuracy. The model training incorporates the ATSS label assignment strategy during the initial stages. In sum, YOLOv6 is particularly well-suited as a behavior detection method for industrial applications.

**YOLOv7-Tiny** [38]: YOLOv7-Tiny, a lightweight network introduced by the YOLOv7 system, features fewer layers and parameters, making it more compatible with GPU devices in specific deployment contexts. Consequently, YOLOv7-Tiny holds promise for application in industrial environments.

### 5.4. Training

The experiments were executed using an Intel(R) Xeon(R) Platinum 8358P CPU boasting 24 GB of RAM, alongside an NVIDIA Tesla 3090 GPU. The software stack utilized PyTorch 1.8.1, Python 3.8, and CUDA 11.1.

For the training phase, we employed the pre-training weights (yolov7.pt) provided by YOLOv7. The stochastic gradient descent (SGD) [51] algorithm was adopted as the optimizer for updating and refining the network model weights. To mitigate model oscillations due to a high initial learning rate, a warm-up strategy was incorporated during training. Within this warm-up phase, the model’s learning rate was gradually increased to reach 0.01. Following the warm-up, the network’s learning rate was dynamically adjusted using the cosine annealing algorithm. Specific experimental parameters were set as follows: batch size of 12, learning rate of 0.01, and weight update factor of 0.0005.

**Figure 9:**
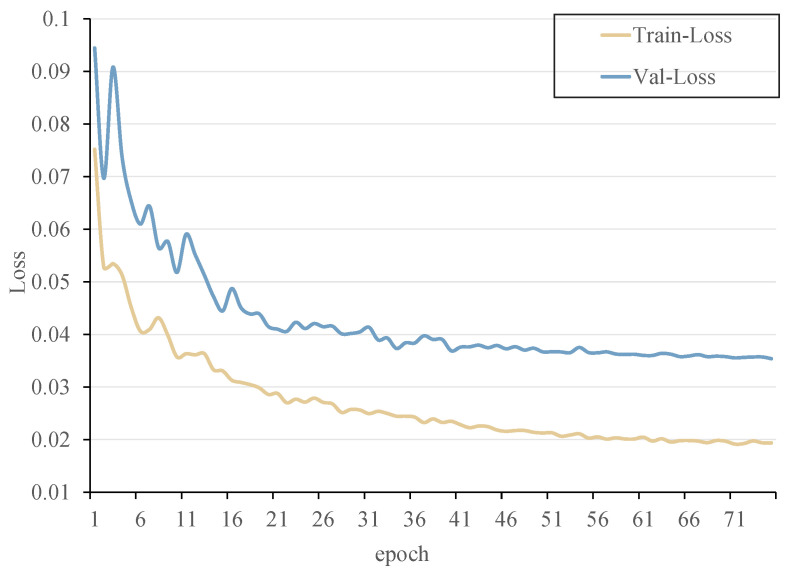
Training and validation losses for students’ learning behavior recognition.

The progress of the detector’s loss was tracked during training, as depicted in Figure 9. Notably, the training and validation losses for the student learning behavior detector converged satisfactorily after 75 rounds.

The observed training progress conclusively establishes that the learned model successfully avoids both overfitting and underfitting, attesting to its optimal suitability for subsequent experiments.

### 5.5. Comparison with YOLOv7 on Individual Learning Behavior Recognition

We conducted empirical research from two perspectives: a comparison with the baseline YOLOv7 in single-class learning behavior recognition and a comparison with six benchmark methods in overall learning behavior recognition. For the sake of convenience in describing, we have named our method the Student Learning Behavior Recognition Framework (SLBRF).

The results of the single-class comparison between our proposed method and the baseline models on the SLB test dataset are provided in Table 4. These results clearly illustrate the superior single-class average precisions (APs) achieved by our proposed method in comparison to the benchmark models. Notably, our method surpasses the YOLOv7 network model on the SLB dataset, showcasing its effectiveness in the domain of student learning behavior recognition.

**Figure 10:**
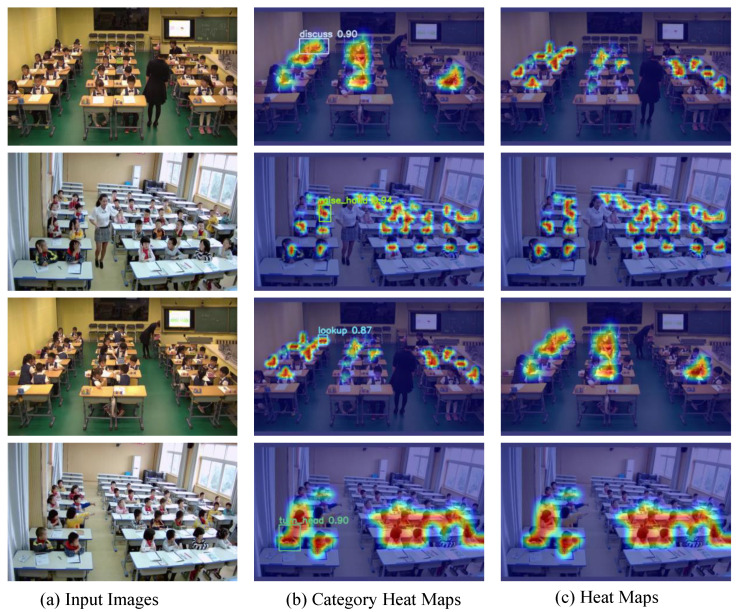
Heat map generated by the GradCAM visualization model on SLB test dataset.

Furthermore, Figure 10 displays the heat map generated using the GradCAM [52] visualization model on the original SLB test set images, highlighting key behavioral features. The prominent orange areas in the heat map indicate the successful localization of relevant image features by the student behavior recognition network. This visualization solidifies the efficacy of our proposed method in accurately identifying and highlighting learned behaviors.

These compelling results underscore the superiority of our proposed method in detecting student learning behaviors, substantiating its efficacy and potential applications in educational contexts.

### 5.6. Comparison with Six Baseline Methods

To further substantiate the performance of our proposed method, a comparison is conducted with YOLOv7-Tiny, the SSD lightweight network, and other prominent classical networks. The comparison experiments employ the same dataset and data configuration, with the results summarized in Table 5.

As evident in Table 5, our proposed method consistently outperforms YOLOv7-Tiny, SSD, YOLOv3-SPP, Faster R-CNN, YOLOv5, and YOLOv6, with a noteworthy minimum improvement of 19% in terms of mAP. These results unequivocally establish the superiority of our proposed method over other mainstream networks. In addition to this, in order to analyze the time cost, inference is performed on RTX 2080TI (11G), 3090 (24G) and 12v 8255 cpu. The results show that our method achieves a good balance between precision and speed.

### 5.7. Ablation Experiments

**Figure 11:**
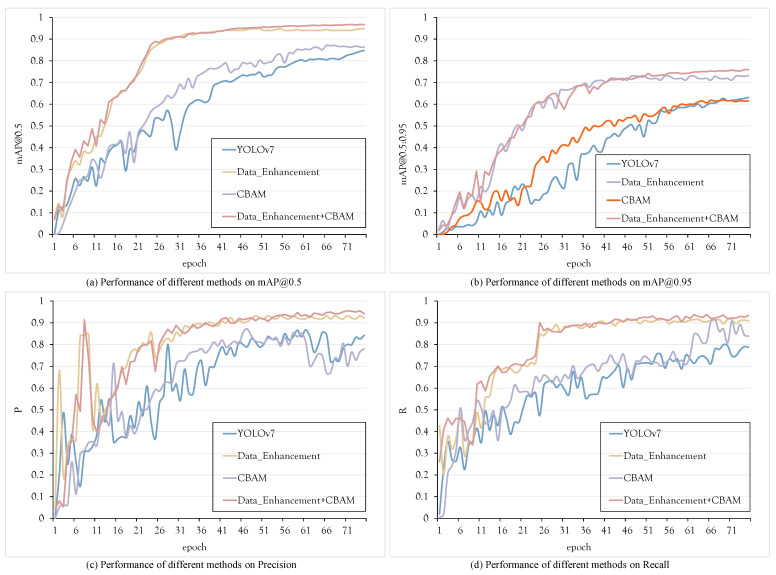
Experimental results of each model on the SLB dataset.

Ablation experiments play a pivotal role in dissecting the impact of network structure modifications on performance. In this section, we present and analyze the outcomes of four essential experiments: YOLOv7, SLBRF with Data Enhancement (SLBRF_DE), SLBRF with the CBAM attention mechanism (SLBRF_CB), and SLBRF with both Data Enhancement and CBAM (SLBRF_DE_CB). The visual training process of these methods on the SLB dataset is depicted in Figure 11, with corresponding training results summarized in Table 6.

**Figure 12:**
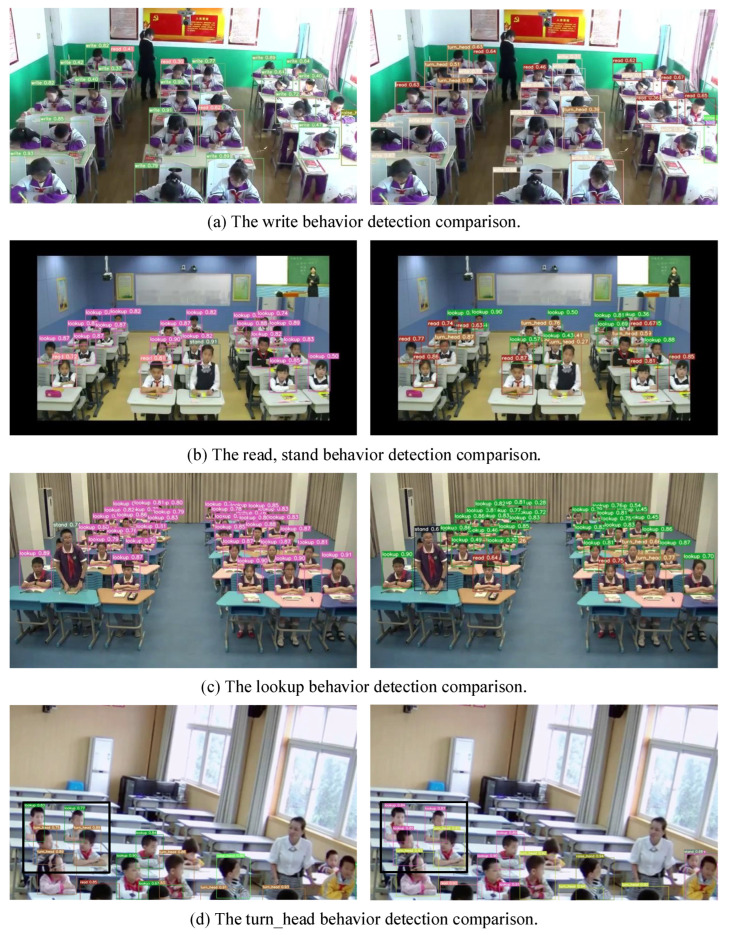
Results of student learning behavior recognition.

In Figure 12, we provide a visual comparison between the proposed method in this paper and the baseline YOLOv7 network’s recognition results in different scenarios. Specifically, the left figure in Figure 12 illustrates the recognition outcomes of the proposed method, while the right figure presents the recognition results of the YOLOv7 baseline.

#### 5.7.1. The Effectiveness of CBAM

The integration of the CBAM attention mechanism exhibited a tangible enhancement in the performance of the student classroom learning behavior recognition network. It led to a 0.7% increase in mAP on the SLB dataset, while concurrently maintaining the number of parameters and FLOPs at the baseline network level. This observation underlines the constructive influence of the CBAM module on enhancing the accuracy of student classroom learning behavior recognition.

#### 5.7.2. The Effectiveness of Data Enhancement

Data augmentation, a crucial technique for mitigating small dataset limitations, not only expanded the dataset to boost the model’s generalization capacity but also increased its adaptability to diverse input data, thereby reinforcing the model’s robustness. The results presented in Table 6 clearly demonstrate an improvement of up to 10.7% in the network’s mAP on the SLB dataset, all without introducing additional computational overhead, as indicated by the steady parameters count and FLOPs.

#### 5.7.3. The Effectiveness of the Model Ensemble

The ultimate recognition outcomes of the integrated model, as presented in Table 4, substantiate its superiority over the baseline model for each category. Improved Average Precision (AP) values were observed across all categories, attesting to the model’s exceptional overall recognition performance. Moreover, Figure 12 provides visual evidence of diverse behaviors within different scenarios. A stark enhancement in recognition accuracy over the baseline model is evident in Figure 12d, where the head-turn behavior, denoted by the black box, is correctly detected, rectifying the previous erroneous outcomes and markedly improving accuracy.

Lastly, the visual representation in Figure 12 provides vivid confirmation of the proposed method’s efficacy in boosting recognition accuracy, especially in challenging scenarios where the YOLOv7 model may encounter difficulties. These findings provide further validation of the proposed method’s superior performance and its capability to accurately detect targets across a variety of real-world scenarios.

#### 5.7.4. The Effectiveness of Attention Mechanism in Learning Behavior Recognition

Given the proven widespread efficacy and performance of attention mechanisms in target recognition, this study extended its exploration to include other attention mechanism modules within the YOLOv7 network. As demonstrated in Table 7, the inclusion of the CBAM module notably amplified the recognition accuracy of the network compared to alternative attention mechanism modules, including CA, SE, and SimAM modules. This outcome emphasizes the CBAM module’s potential in boosting recognition performance.

In Table 7, a comprehensive comparison is presented between networks utilizing different attention mechanism modules. The metrics evaluated include mAP, precision, recall, FLOPs, parameters (Params), and model weight size. The results demonstrate the following:

- YOLOv7 achieves an mAP of 84.8%, with a precision of 86.24% and a recall of 77.8%. It has 105.2 billion FLOPs, $3722.3\times 10^{4}$ parameters, and a model weight size of 71.4 million;
- Incorporating the SE module in YOLOv7 leads to a slight decrease in mAP (84.2%), precision (83.4%), and recall (78.4%), while maintaining the same FLOPs and increasing the parameters to $3723.5\times 10^{4}$ and model weight size to 113 million;
- Utilizing the CA module in YOLOv7 results in a further decrease in mAP (82.0%), precision (82.81%), and recall (77.83%). The FLOPs remain the same, while the parameters decrease to $3716.7\times 10^{4}$ and the model weight size remains at 71.3 million;
- Applying the SimAM module in YOLOv7 leads to a slight improvement in mAP (84.6%), but precision (81.7%) and recall (84.8%) show mixed changes. The FLOPs and parameters remain consistent, and the model weight size remains at 71.3 million;
- Incorporating the CBAM module (proposed in this paper) in YOLOv7 results in the highest mAP of 85.5% (an increase from the baseline). The precision is 79.73%, and the recall reaches 88.02%. The FLOPs and parameters remain consistent with YOLOv7, while the model weight size remains at 71.4 million.

In summary, the inclusion of the CBAM attention mechanism in YOLOv7 proves to be the most effective in terms of improving recognition accuracy, surpassing other attention mechanism modules such as SE, CA, and SimAM. It achieves the highest mAP, precision, and recall while maintaining a comparable number of parameters, FLOPs, and model weight size. These findings highlight the superiority of the proposed method in this paper in terms of attention mechanisms for target recognition.

## 6. Conclusions

In this study, we have presented an effective student learning behavior detector based on the YOLOv7 network, enabling accurate recognition of classroom learning behaviors. We have addressed the challenge of limited data samples through the strategic application of transfer learning and data augmentation techniques. Additionally, by integrating the CBAM attention mechanism into the YOLOv7 feature detection network, we have amplified its ability to extract vital information about students’ learning behaviors, thereby enhancing feature recognition accuracy in classroom settings. The experimental results substantiate the superiority of our approach over YOLOv7-Tiny, SSD lightweight networks, and other prominent classical networks in the context of student learning behavior recognition. We have achieved noteworthy advancements in the precise identification and categorization of various learning behaviors exhibited by students in the classroom.

However, it is essential to acknowledge the limitations of our study. Notably, the dataset employed in our experiments includes behaviors that should have been more rigorously defined and labeled. This limitation may have introduced some ambiguity and noise during both training and evaluation. Moreover, we recognize that the current network model we have proposed, while powerful, is relatively large, which may present performance challenges when deployed on embedded devices with limited GPU resources. To overcome this limitation, we acknowledge the necessity of developing lightweight yet high-performing models tailored for real-time recognition in resource-constrained settings. Our upcoming research efforts will be directed towards substantial improvements in these areas.

In future studies, we are committed to addressing this issue by refining the dataset and providing more precise behavior definitions, ensuring higher-quality training data. Additionally, due to objective factors such as data acquisition equipment and acquisition angles, the dataset we constructed is not sufficiently large, resulting in lower data resolution. Therefore, we intend to expand a high-quality dataset for future research. In order to enhance users’ personal privacy and data security, we plan to introduce federated learning in future model deployments. This initiative will ensure the adequate protection of students’ personal data. Simultaneously, we will further optimize the model by moving the data acquisition and processing phases to the client side, avoiding the transfer of students’ image data to the server side, thereby reducing the potential risk of data transmission. This approach not only helps protect users’ personal privacy, but also reduces the need for server-side data storage, improving the overall system security.

Introducing computer vision technology into modern education evaluation system can provide many useful tools and methods for education and teaching staff to improve the quality, efficiency, and fairness of education evaluation. At the same time, our results show that the use of visual techniques has great potential for classroom analysis.

## Figures and Tables

**Table 1:** Comparison of student learning behavior recognition methods based on deep learning.

Method	Technique	Performance
Faster R-CNN [24]	1. Proposal of an improved Faster R-CNN model; 2. Introduction of a scale-aware detection head to handle scale variations; 3. Feature fusion strategy to detect low-resolution behaviors; 4. Use of Online Hard Example Mining (OHEM) to address class imbalances.	Experimental results on real corpus show that the performance of the proposed method is improved compared with the baseline method.
SSD [25]	1. Data enhancement techniques were applied to expand the dataset; 2. The improved model showed better feature extraction ability and small target recognition accuracy compared to the native SSD model.	The improved SSD model achieves high recognition accuracy and provides technical support for intelligent management and teaching in universities.
YOLOv3 [26]	The researchers used the YOLOv3 algorithm for face recognition and prediction of student behavior.	The proposed method offers a reasonable pace of identification and positive outcomes for measuring student interest based on observable actions in the classroom.
YOLOv5 [27]	1. Proposal of a classroom behavior detection algorithm using an improved YOLOv5 model; 2. Combination of feature pyramid structure and weighted bidirectional feature pyramid network; 3. Addition of spatial and channel convolutional attention mechanism; 4. Improvement of non-maximum suppression using distance-based intersection ratio.	The average precision of the algorithm on the self-built dataset is 89.8%, and the recall rate is 90.4%.

**Table 2:** Symbols and notations.

Number	Notation	Description
1	(x,y,w,h,o)	The position coordinates and background of the marker box
2	**MLP**	Multi-Layer Perceptron Calculator
3	$W_{x}$	MLP layer *x* parameters
4	**F**	Feature Map
5	⊗	Pixel-level multiplication
6	$M_{c}\left(F\right)$	Channel Attention maps
7	$M_{s}\left(F\right)$	Spatial Attention Maps
8	$f^{7\times 7}$	7 × 7 convolution

**Table 3:** Details of the SLB dataset.

Number	Classes	Num of Labels	Train	Val	Test
1	write	1025	452	491	82
2	read	1075	810	139	126
3	lookup	5725	3620	1656	449
4	turn_head	1025	748	117	160
5	raise_hand	725	561	82	82
6	stand	94	50	30	14
7	discuss	242	172	50	20

**Table 4:** Single-class AP results on the test set. The bold means the best performance.

Classes	YOLOv7 (%)	Proposed Method (%)
write	87.2	**98.7**
read	71.3	**93.0**
lookup	94.4	**98.6**
turn_head	84.7	**96.4**
raise_hand	95.0	**99.1**
stand	78.2	**93.0**
discuss	96.6	**98.1**
Total	84.8	**96.7**

**Table 5:** Comparison of performance and speed of different networks. The bold means the best performance. The underline means the second best performance.

Methods	mAP@0.5 (%)	AP50 (%)	FPS (f/s)		
			2080Ti 11G*1	3090 24G*1	**12v 8255C (CPU)**
SSD	65.7	43.0	10.5	18.0	0.1
Faster R-CNN	68.9	49.6	30.6	54.6	0.1
YOLOv3-SPP	63.1	43.0	80.6	**95.2**	3.9
YOLOv5	76.9	54.9	**97.9**	92.5	1.8
YOLOv6	77.7	52.9	62.8	89.1	4.7
YOLOv7-Tiny	54.3	34.8	86.2	81.3	**12.8**
SLBRF(Our Method)	**96.7**	**75.8**	80	84.8	2.7

**Table 6:** Performance comparison of different models on the SLB dataset. The bold means the best performance.

Model	mAP@0.5 (%)	mAP@0.5:0.95 (%)	Params (10^4^)	FLOPs (G)	Weight (M)
YOLOv7	84.8	62.7	3722.3	105.2	71.4
SLBRF_CB	85.5	-	**3721.2**	105.2	71.4
SLBRF_DE	95.5	73.6	3722.7	105.2	71.4
SLBRF_DE_CB(ours)	**96.7**	**75.9**	**3721.2**	**105.0**	71.4

**Table 7:** Comparison of networks using different attention mechanism modules.

Model	mAP (%)	Precision (%)	Recall (%)	FLOPs (G)	Params (10^4^)	Weight (M)
YOLOv7	84.8	86.24	77.8	105.2	3722.3	71.4
SLBRF_SE	84.2 ↓	83.4	78.4	105.2	3723.5	113
SLBRF_CA	82.0 ↓	82.81	77.83	105.0	3716.7	71.3
SLBRF_SimAM	84.6 ↓	81.7	84.8	105.0	3721.0	71.3
SLBRF_CBAM (ours)	85.5 ↑	79.73	88.02	105.0	3721.2	71.4

## Data Availability

Data will be made available on reasonable request.

## Abbreviations

The following abbreviations are used in this manuscript:

FPN	Feature Pyramid Network
PAN	Path Aggregation Network
IoT	Internet of Things
SOTA	State-of-the-Art
CNNs	Convolutional Neural Networks
CBAM	Convolutional Block Attention Module
CAM	Channel Attention Module
SAM	Spatial Attention Module
SSD	Single Shot MultiBox Detector
DBN	Deep Belief Network
YOLO	You Only Look Once
ViT	Vision Transformer
DETR	Detection Transformer
W-MSA	Windowed Multihead Self-Attention
SW-MSA	Sliding-Window Multihead Self-Attention
MLP	Multilayer Perceptron
LN	Layer Normalization
CARAFE	Content-Aware Reassembly of Features
mAP	Mean Average Precision
RPN	Region Proposal Network
CBL	Convolutional Block Layer
PAN	Path Aggregation Network
ELAN	Efficient Local Attention Network
CAT	Category-aware Transformation
